# Mantle wedge diapirs detected by a dense seismic array in Northern Taiwan

**DOI:** 10.1038/s41598-021-81357-7

**Published:** 2021-01-15

**Authors:** Cheng-Horng Lin, Min-Hung Shih, Ya-Chuan Lai

**Affiliations:** 1 Institute of Earth Sciences, Academia Sinica, Nankang, PO Box 1-55, Taipei, Taiwan; 2 National Center for Research on Earthquake Engineering, National Applied Research Laboratories, Taipei, Taiwan; 3 Taiwan Volcano Observatory at Tatun, Taipei, Taiwan; 4 Department of Geosciences, National Taiwan University, Taipei, Taiwan

**Keywords:** Natural hazards, Solid Earth sciences

## Abstract

It is conventionally believed that magma generation beneath the volcanic arc is triggered by the infiltration of fluids or melts derived from the subducted slab. However, recently geochemical analyses argue the arc magma may be formed by mélange diapirs that are physically mixed by sediment, altered oceanic crust, fluids, and mantle above the subducted slab. Further numerical modeling predicts that the mantle wedge diapirs have significant seismic velocity anomalies, even though these have not been observed yet. Here we show that unambiguously later P-waves scattered from some obstacles in the mantle wedge are well recorded at a dense seismic array (Formosa Array) in northern Taiwan. It is the first detection of seismic scattering obstacles in the mantle wedge. Although the exact shape and size of the scattered obstacles are not well constrained by the arrival-times of the later P-waves, the first order approximation of several spheres with radius of ~ 1 km provides a plausible interpretation. Since these obstacles were located just beneath the magma reservoirs around depths between 60 and 95 km, we conclude they may be mantle wedge diapirs that are likely associated with magma generation beneath active volcanoes.

## Introduction

The strongest and most frequent volcanic eruptions on Earth usually take place in volcanic arcs, where energetic magma reservoirs are often observed in the overlying crust above the subducted slab. It is conventionally believed that those magmas that gradually accumulate within the reservoirs are first generated in the mantle wedge due to infiltration of fluids and melts derived from subducted oceanic sediments or crust, and then ascend into the reservoirs^1–3^. However, such a conventional concept of magma generation beneath volcanic arcs has been challenged by some numerical models^4–6^ as well as geochemical observations^7–10^. A novel model has proposed that arc-like magmas might be formed by mélange diapirs directly ascending from the slab-top. Those mélange diapirs, which are hybrid rocks composed of blocks of altered oceanic crust and sediments mixed with mantle-wedge peridotite, may rise from the slab-mantle interface. The petrological-thermomechanical model further suggests those mélange diapirs have significant characteristics of seismic anomalies^5^.

However, there has been no direct seismic evidence that shows mélange diapirs in the past, even though many seismic images within mantle wedges have been captured across the world^11–16^. Among these seismic images, the most typical observations in mantle wedges are some ambiguous low-velocity zones without any significant seismic boundary, which are likely linked to partial melting above the subducted slab. If diapirs do exist, then the absence of seismic evidence for mélange diapirs might be attributed to the poor resolution of seismic images obtained from seismic tomography or other seismic techniques. In other words, the mélange diapirs might be too small to be detected due to the limitation of seismic station coverage as well as the particular geometry of seismicity along the subduction zone.

**Figure 1:**
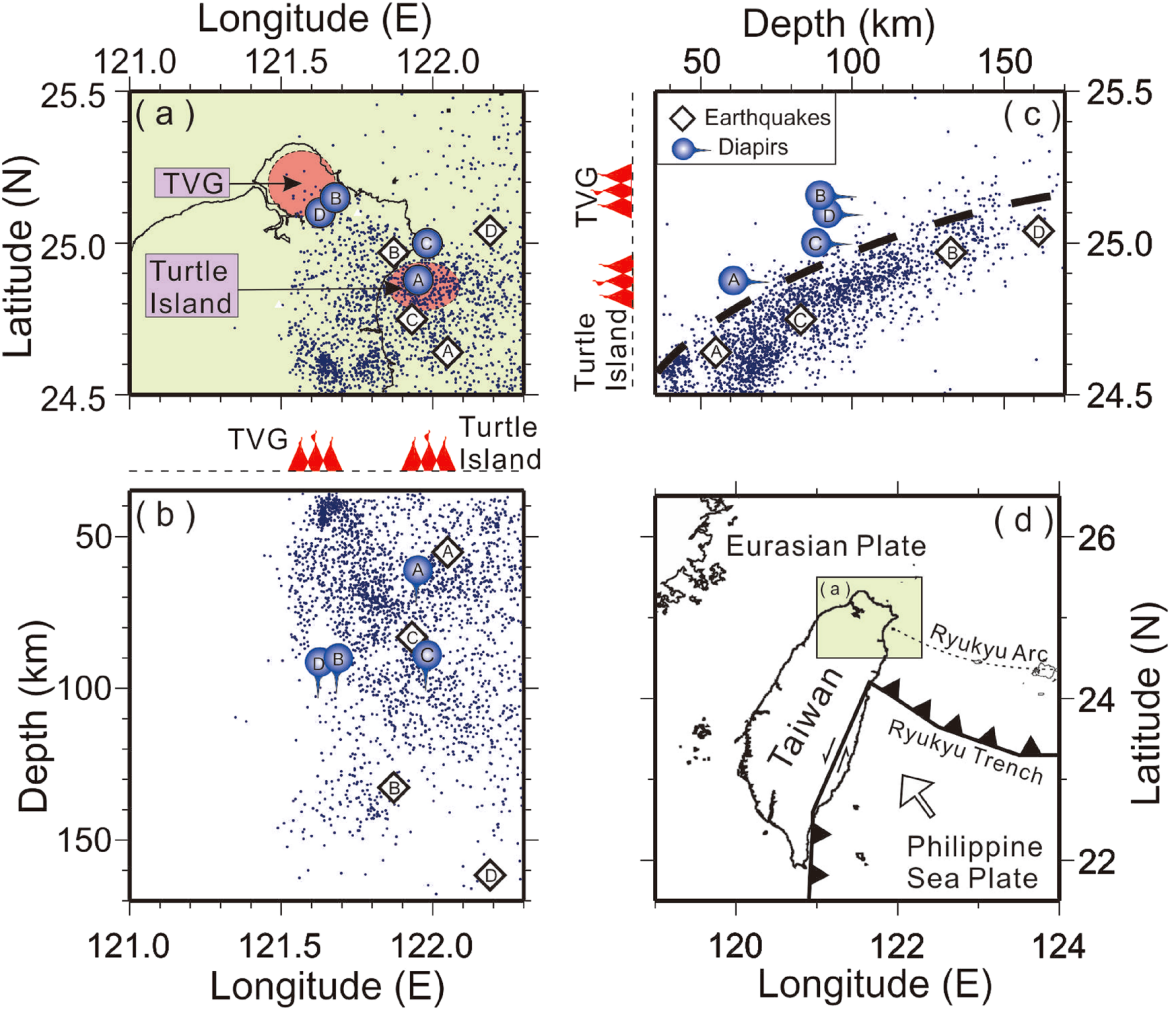
Locations of large earthquakes (diamonds), background seismicity (small dots), diapirs (blue lightbulbs or circles) and two active volcanoes (red areas) are projected on the map (**a**) and two depth profiles (**b**) and (**c**), respectively. The general tectonics in the Taiwan area is plotted in (**d**). This figure was created by Generic Mapping Tools (GMT version 4.5.2; URL: gmt.soest.hawaii.edu).

Taiwan is located at the convergent boundary between the Philippine Sea plate and Eurasian plate (Fig. 1). The Philippine Sea plate is moving northwestward and subducting beneath the Eurasian plate along the Ryukyu trench^17^. Like many ordinary subduction systems, plenty of active volcanoes develop along the Ryukyu volcanic arc from Kyusyu, southern Japan, to northern Taiwan^18,19^. Taiwan is located just at the westernmost end of the Ryukyu volcanic arc^20–22^, where two active volcanoes have been identified^23–28^. One is the Tatun Volcano Group (TVG) nearby the Taipei metropolitan area, and the other is Kueishantao (or Turtle Island), named for its shape, offshore from the Ilan plain of northeastern Taiwan. Despite the absence of eruption records in human history, both volcanoes have been recognized as active based on a variety of recent observations including Helium isotope analyses^25^, dating of volcanic ashes^26^, seismic detection of magma reservoirs^27,28^, and some other interesting seismic activities such as heartbeat-like seismicity^29^, VLP tremors^30^, infrasonic signals^31^, and many others^32,33^. Thus, more detailed investigation of both volcanoes becomes very important in Taiwan since potential volcanic impacts cannot be totally excluded in the future.

**Figure 2:**
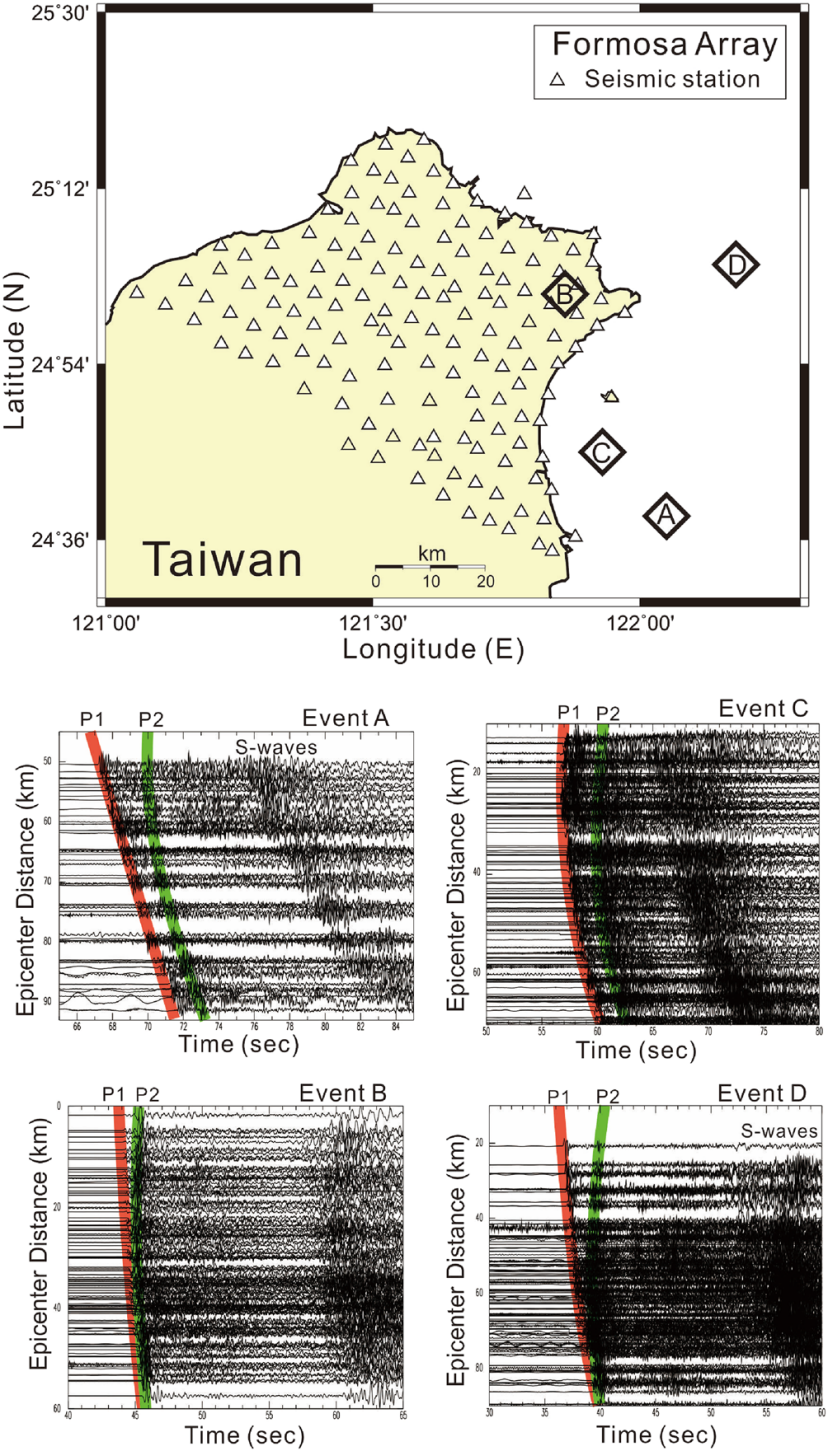
Locations of Formosa Array (the upper panel) and strong later P-waves (P2) are recorded at the vertical seismograms generated by 4 earthquakes (Events A, B, C and D shown at the lower panels) from the subduction zone beneath northern Taiwan. This figure was created by Generic Mapping Tools (GMT version 4.5.2; URL: gmt.soest.hawaii.edu) and SAC software (version 101.5; URL: ds.iris.edu).

For improving seismic images of magma reservoirs as well as other subsurface structures in the northern part of Taiwan, a high-density broadband seismic array (Formosa Array) has been deployed since 2018 (Fig. 2). Despite the fact that magma reservoirs beneath both the TVG and Turtle Island have been detected by seismic observations of both S-wave shadows and P-wave delays from the dense seismic stations focused on two volcanic areas^27,28^, the exact geometry of magma reservoirs are still poorly obtained. The Formosa Array is designed to examine the existence of magma reservoirs and to improve seismic images beneath volcanic areas, with the installation of 146 broadband seismic stations in the northern Taiwan area. This dense seismic array with station spacing distances of ~ 5 km evenly covers the northernmost part of Taiwan, an area of ~ 50 km × ~ 80 km. Each seismic station is equipped with a broadband seismometer (Meridian Compact PH by Nanometrics) installed into a shallow borehole at a depth of ~ 200 cm below the surface. Seismic data are continuously recorded with a sampling rate of 100 Hz and then transmitted in real-time by either telephone cable or wireless radio to the Institute of Earth Sciences, Academia Sinica, and the Taiwan Volcano Observatory at Tatun (TVO) in Taipei, Taiwan.

## Results

### Strong later P-waves

**Figure 3:**
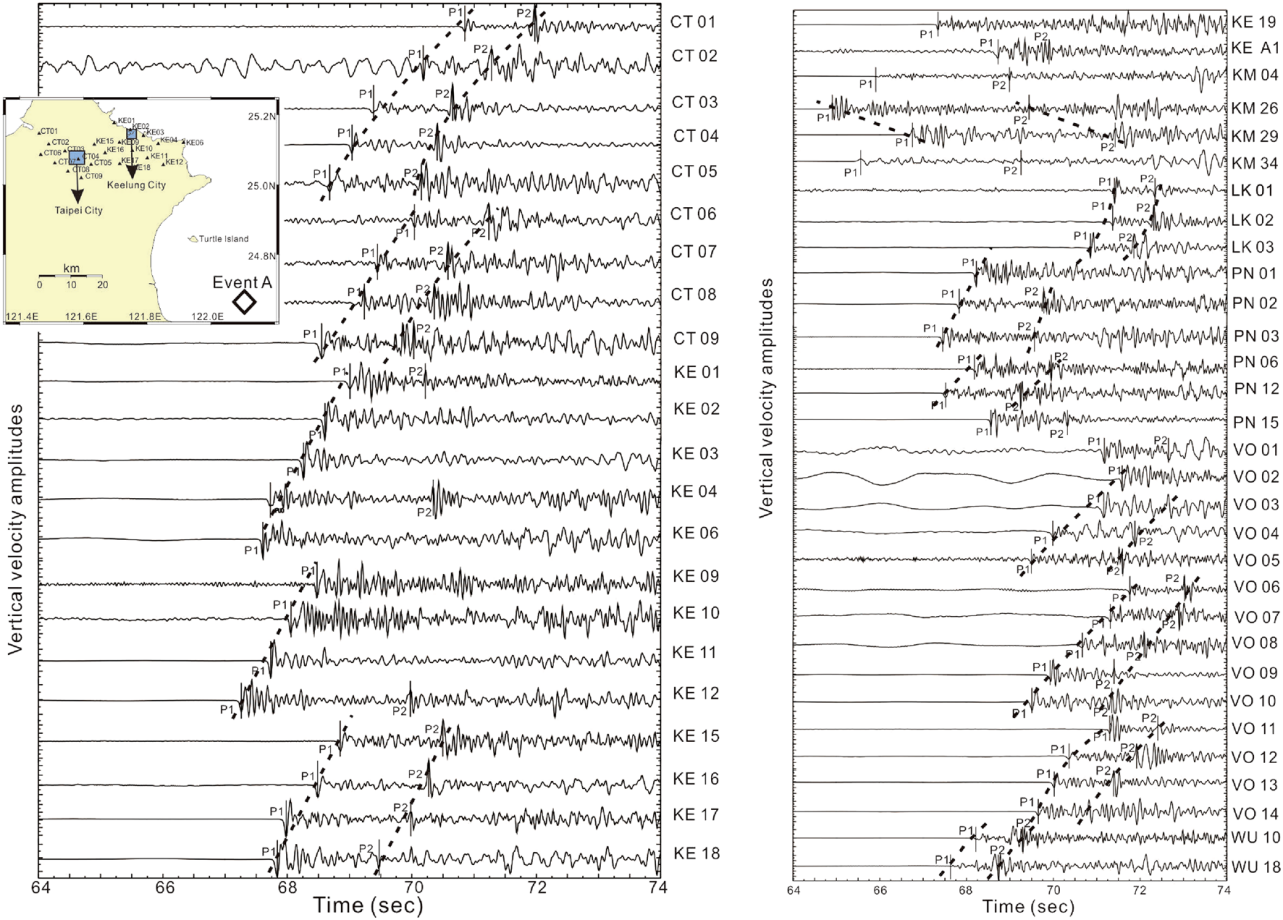
Arrivals of P1 and P2 (vertical bars) generated by Event A and recorded at the Formosa Array. The dashed lines mark the alignment of P1 and P2 arrivals at the adjacent stations. The insert map shows locations of seismic stations (triangles) and Event A (diamond). This figure was created by SAC software (version 101.5; URL: ds.iris.edu).

Four earthquakes (Fig. 2, Table 1) that have clear later P-waves recorded at the Formosa Array were chosen after we carefully examined all deep earthquakes (depth > 50 km) beneath the northern Taiwan area since 2018. The later P-waves were recorded at seismic stations less than a few of seconds after the direct P-waves. In fact, some later P-waves can also be recognized from other earthquakes but these are not discussed below in detail because the amplitudes of later P-waves are not very clear for reading the arrivals. For Events A, B, C, and D listed in Table 1, it is surprising to note that strong later P-waves are unambiguously identified at almost all seismic stations in the Formosa Array (Fig. 2). In addition to the direct P-waves (P1) from the hypocenter to stations, the later P-wave (P2) arrivals are coincidently found from four earthquakes whose locations are distributed at different depths along the subduction zone (Fig. 1c and Table 1). In general, it is unusual to see such a later P-wave (P2) because most ray-paths from local deep earthquakes propagate through the upper mantle, which is too homogeneous to reflect or scatter seismic waves. For instance, the P1 and P2 arrivals from Event A can be directly identified at each individual station because most seismic amplitudes of both P1 and P2 are comparable (i.e., Fig. 3). In addition to the individual station, the consistent energy of P2 arrivals can be recognized from seismograms at adjacent stations within distances of ~ 5 km. In other words, P2 arrivals can be well aligned in the dense seismic array even at the metropolitan area, such as Taipei City, where background noises are usually strong. In general, the delay times between P1 and P2 vary significantly with hypocentral depth. For Events A and C, whose depths are shallower than 90 km, the delay times between P1 and P2 can reach up to 2—4 s. For Events B and D, whose depths are deeper than 130 km, most of the delay times between P1 and P2 are less than 1 s. Those travel-time features between P1 and P2 imply that some scattering obstacles or reflection boundaries may exist within the mantle wedge.

**Table 1:** Earthquake and diapir parameters.

No	Earthquakes					Diapirs		
	Longitude	Latitude	Depth (km)	M_L_	Date	Longitude	Latitude	Depth (km)
Event A	122.05°E	24.64°N	55	4.3	2018/07/19	121.95°E	24.88°N	61
Event B	121.86°E	25.02°N	138	5.3	2019/03/12	121.68°E	25.15°N	90
Event C	121.93°E	24.75°N	83	4.9	2019/03/19	121.98°E	25.00°N	88
Event D	122.18°E	25.07°N	152	5.0	2019/06/29	121.63°E	25.10°N	92

In fact, for Event B, a preliminary analysis based on two-dimensional ray-tracing modeling had suggested that P2 might be reflected from a dipping plane around the depths of 80–110 km within the mantle wedge^34^. But such a simple dipping plane cannot explain the other P2 arrivals recorded at seismic stations away from the 2-D vertical profile. The fact that P2 is recorded at all seismic stations of the Formosa Array in northern Taiwan indicates that seismic energy is likely scattering from different azimuths. Thus, instead of a simple dipping plane, a 3-D obstacle might be more suitable for explaining seismic energy scattering in all incident directions.

**Figure 4:**
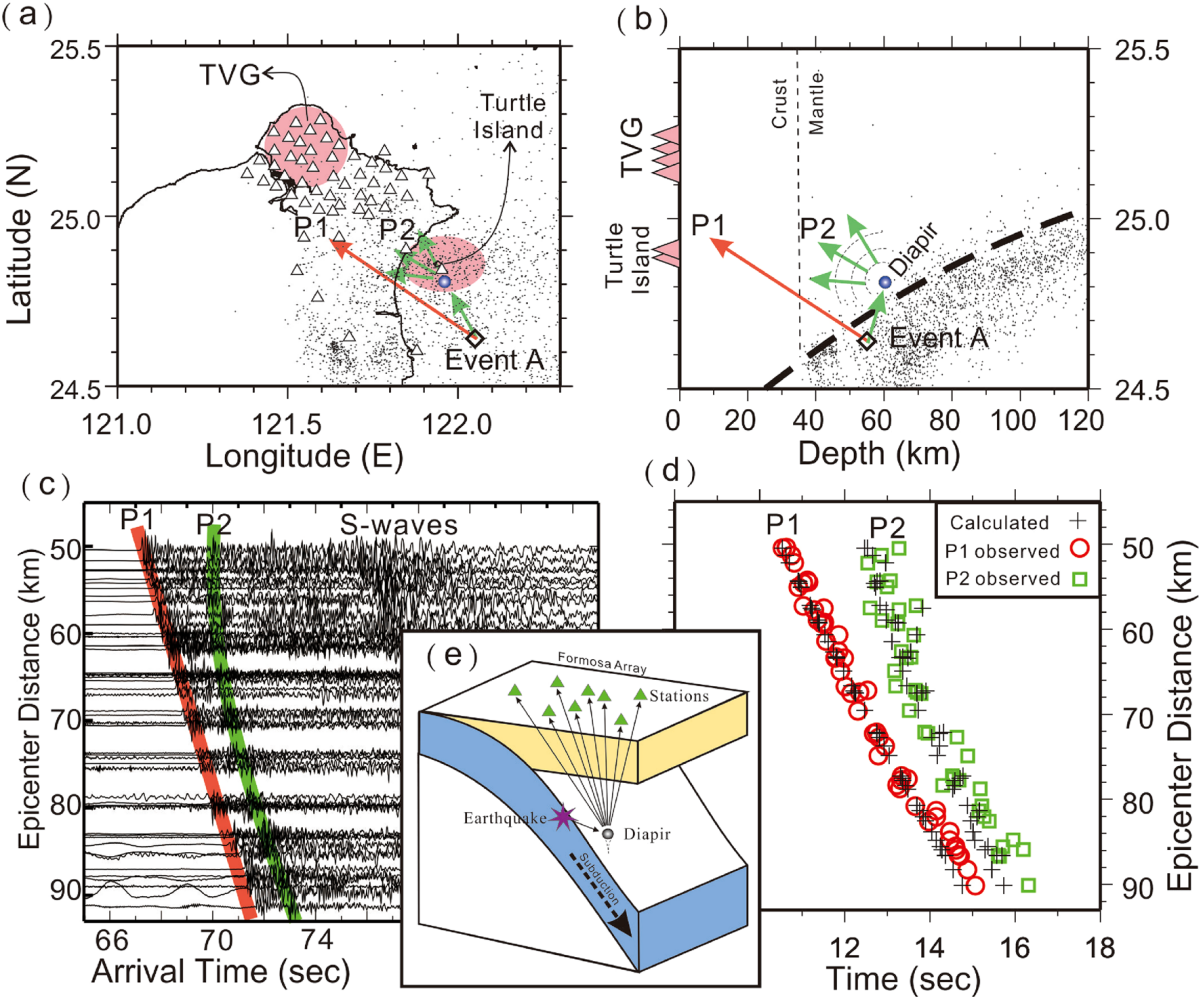
Locations of seismic stations (triangles), Event A (diamond) and diapir (blue circle) on the map (**a**) and depth profile (**b**). Locations of two active volcanoes, the Tatun volcano group (TVG) and Turtle Island are marked in pink. (**c**) The arrivals of P1 and P2 are, respectively, marked in red and green on the vertical seismograms plotted with epicentral distances. (**d**) The comparison between the observed arrivals of P1 (red circles) and P2 (green squares), and their calculated arrivals (pluses). (**e**) A schematic plot showing the ray-paths scattered from the diapir in the mantle wedge. This figure was partially created by Generic Mapping Tools (GMT version 4.5.2; URL: gmt.soest.hawaii.edu) and SAC software (version 101.5; URL: ds.iris.edu).

**Figure 5:**
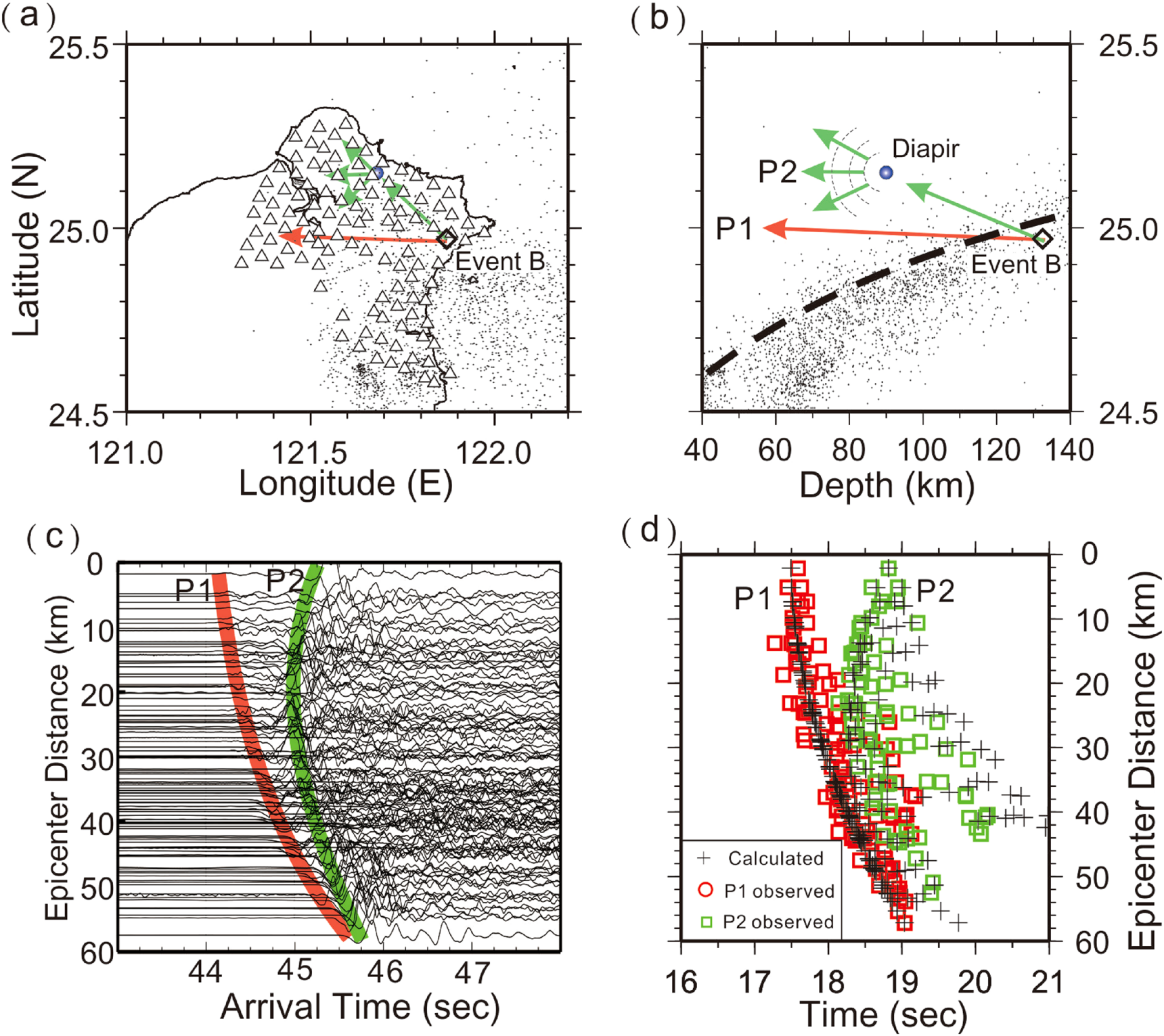
Locations of seismic stations (triangles), Event B (diamond) and diapir (blue circle) on the map (**a**) and depth profile (**b**). (**c**) The arrivals of P1 and P2 are, respectively, marked in red and green on the vertical seismograms plotted with epicentral distances. (**d**) The comparison between the observed arrivals of P1 (red circles) and P2 (green squares), and their calculated arrivals (pluses). This figure was partially created by Generic Mapping Tools (GMT version 4.5.2; URL: gmt.soest.hawaii.edu) and SAC software (version 101.5; URL: ds.iris.edu).

**Figure 6:**
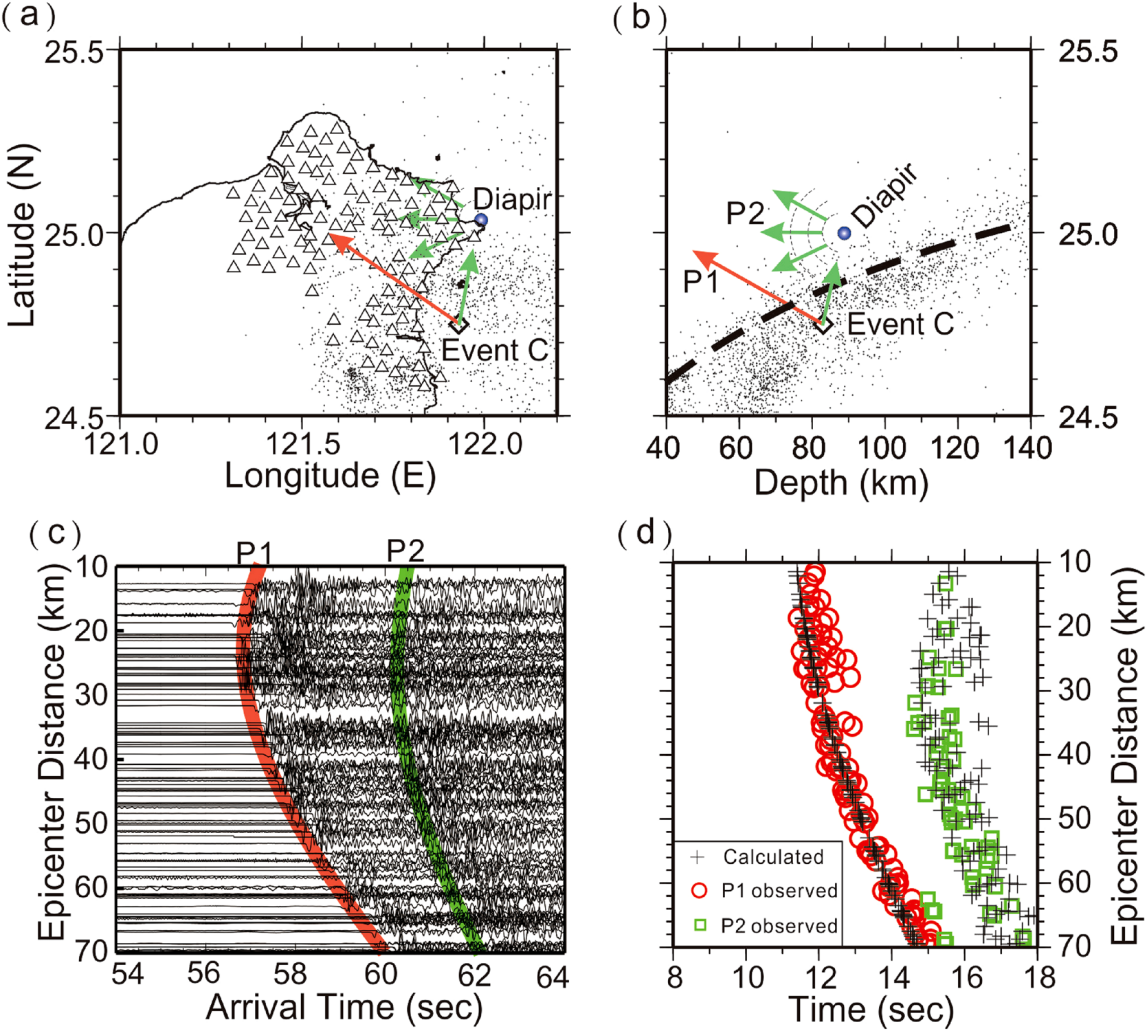
Locations of seismic stations (triangles), Event C (diamond) and diapir (blue circle) on the map (**a**) and depth profile (**b**). (**c**) The arrivals of P1 and P2 are, respectively, marked in red and green on the vertical seismograms plotted with epicentral distances. (**d**) The comparison between the observed arrivals of P1 (red circles) and P2 (green squares), and their calculated arrivals (pluses). This figure was partially created by Generic Mapping Tools (GMT version 4.5.2; URL: gmt.soest.hawaii.edu) and SAC software (version 101.5; URL: ds.iris.edu).

**Figure 7:**
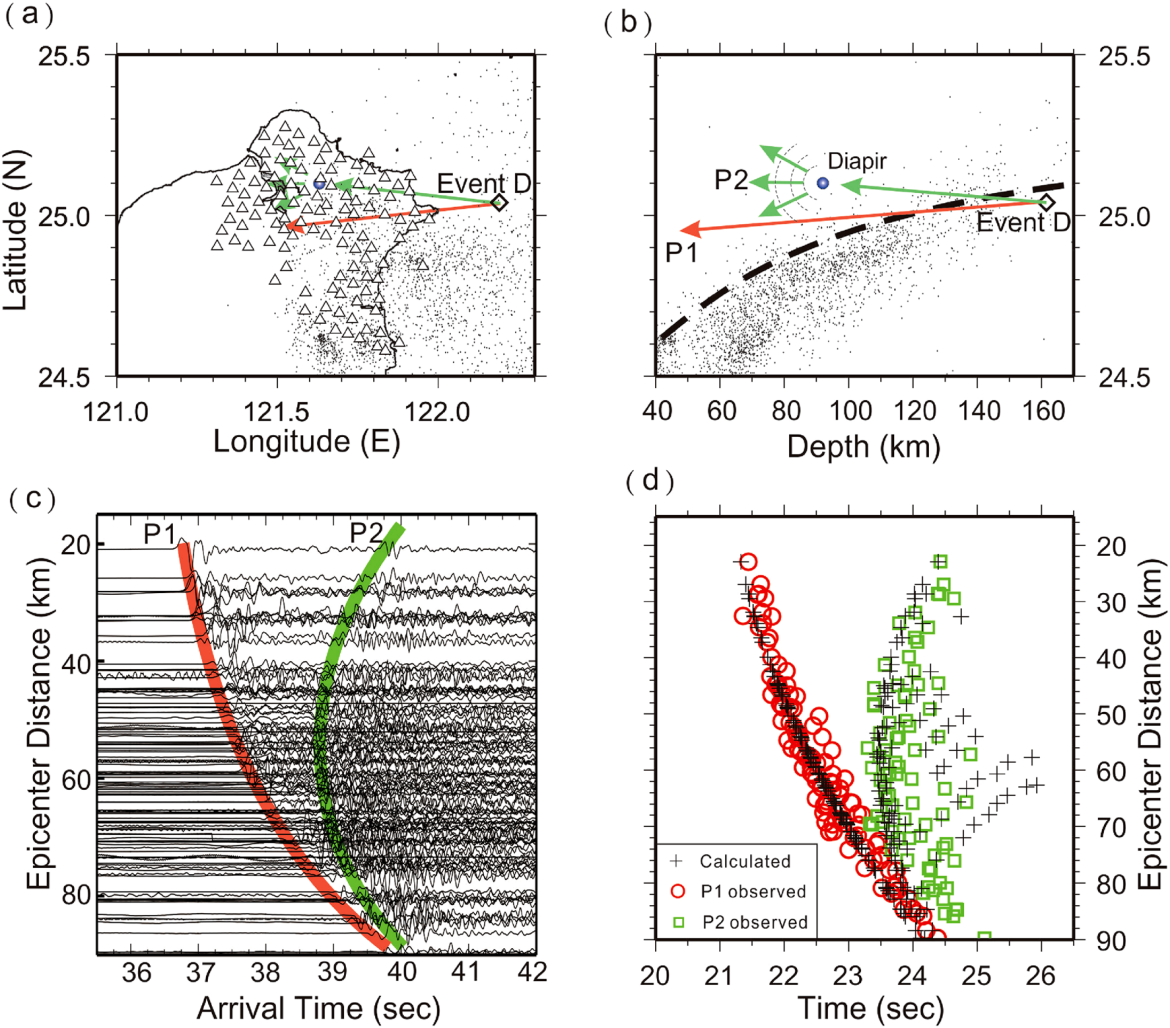
Locations of seismic stations (triangles), Event D (diamond) and diapir (blue circle) on the map (**a**) and depth profile (**b**). (**c**) The arrivals of P1 and P2 are, respectively, marked in red and green on the vertical seismograms plotted with epicentral distances. (**d**) The comparison between the observed arrivals of P1 (red circles) and P2 (green squares), and their calculated arrivals (pluses). This figure was partially created by Generic Mapping Tools (GMT version 4.5.2; URL: gmt.soest.hawaii.edu) and SAC software (version 101.5; URL: ds.iris.edu).

To examine possible locations of the scattering obstacles beneath northern Taiwan, we apply a trial and error method to fit the calculated travel-times of both P1 (direct waves) and P2 (scattered waves) with the observations. Assuming a simplified 1D velocity model of the uppermost mantle and overlying crust, the travel times of both direct and scattering P-waves are calculated for any possible scattering obstacles beneath northern Taiwan. For successfully producing seismic energy at almost any incident direction, we simply assume that the 1st order approximation of the obstacle is a sphere with a radius of ~ 1 km based on the scaling analysis of instability growth rates^35^. Thus, the seismic energy propagating to the sphere obstacle will be scattered into all directions^36–38^ because the radius of the spherical obstacle is much less than the wavelength (~ 8 km) of P-wave, by given its period of ~ 1.0 s and velocity of ~ 8 km/s. The detailed algorithm and data process are described in the Method section. The results below suggest several sphere-like obstacles in the mantle wedge around the depths between 60 and 95 km may exist just beneath two active volcanoes (Turtle Island and the TVG) in the northern Taiwan area (Figs. 4, 5, 6, 7).

### Scattering obstacles beneath the Turtle Island

For Event A, the sphere-like scattering obstacle that fits both P1 and P2 arrivals is located just beneath Turtle Island at a depth of 61 km within the mantle wedge and above the subducted slab (Fig. 4a,b). At first, the calculated P1 arrivals recorded at Formosa Array (Fig. 4c) fit well with the observations (Fig. 4d) because they are simply dependent on the hypocentral distances. On the other hand, the P2 arrivals are sensitive to both the scattering obstacle and the hypocenter. Most P2 arrivals basically follow a hyperbolic curve on the travel-time table (Fig. 4d) because the epicenter is located outside the seismic array. Although there are some small differences (less than 1 s) between the calculations and observations, the general patterns between them are comparable. Outliers between the calculations and observations could be attributed to not only a simplified 1D model and earthquake location for calculating travel-times, but also possible reading errors produced by unexpected noise at each seismic station. A schematic plot of ray-paths scattered from the sphere-like obstacle in the mantle wedge is shown at Fig. 4e.

The scattering obstacle of Event C is also identified nearby Turtle Island, but at a depth of 88 km (Fig. 5). Although its depth is greater than that obtained from Event A, both are found just on top of the subducted slab with a distance of less than 20 km (Fig. 1). Again, most P2 arrivals basically follow a hyperbolic curve on the travel time table because the scattering obstacle and earthquake are located outside the Formosa Array (i.e., Fig. 4). The travel-time fit for both P1 and P2 is generally acceptable without considering complicated structures and possible reading errors at seismograms.

### Scattering obstacles beneath the TVG

The scattering obstacles of Events B and D are grouped beneath the TVG at depths of ~ 90 km (Figs. 6 and 7), which are only around 20–30 km away from the subducted slab (Fig. 1). Since both obstacles are detected inside the Formosa Array, the travel-time differences between P1 and P2 strongly change with epicentral distance and then become more complicated (Fig. 6). Most P2 arrivals also follow a hyperbolic curve on the travel time table, but it is interesting to note that some P2 arrivals on the travel-time table become more scattered at the same epicentral distances (Figs. 6 and 7). This scattering phenomenon is attributed to the fact that the obstacle is located in the middle of the Array. In other words, at the same epicentral distances, the delay time between P1 and P2 becomes significant if P2 largely scatters backward. Although some differences between the calculations and observations might reach up to ~ 1 s, the general patterns between them are still comparable if we consider possible uncertainties from both calculating 1D velocity model and reading errors again. Even so, the small number of outliers (less than 1.0 s) at some stations might only result in an uncertainty of several kilometers for locating the obstacle.

### Sensitivity of locating scatter obstacles

**Figure 8:**
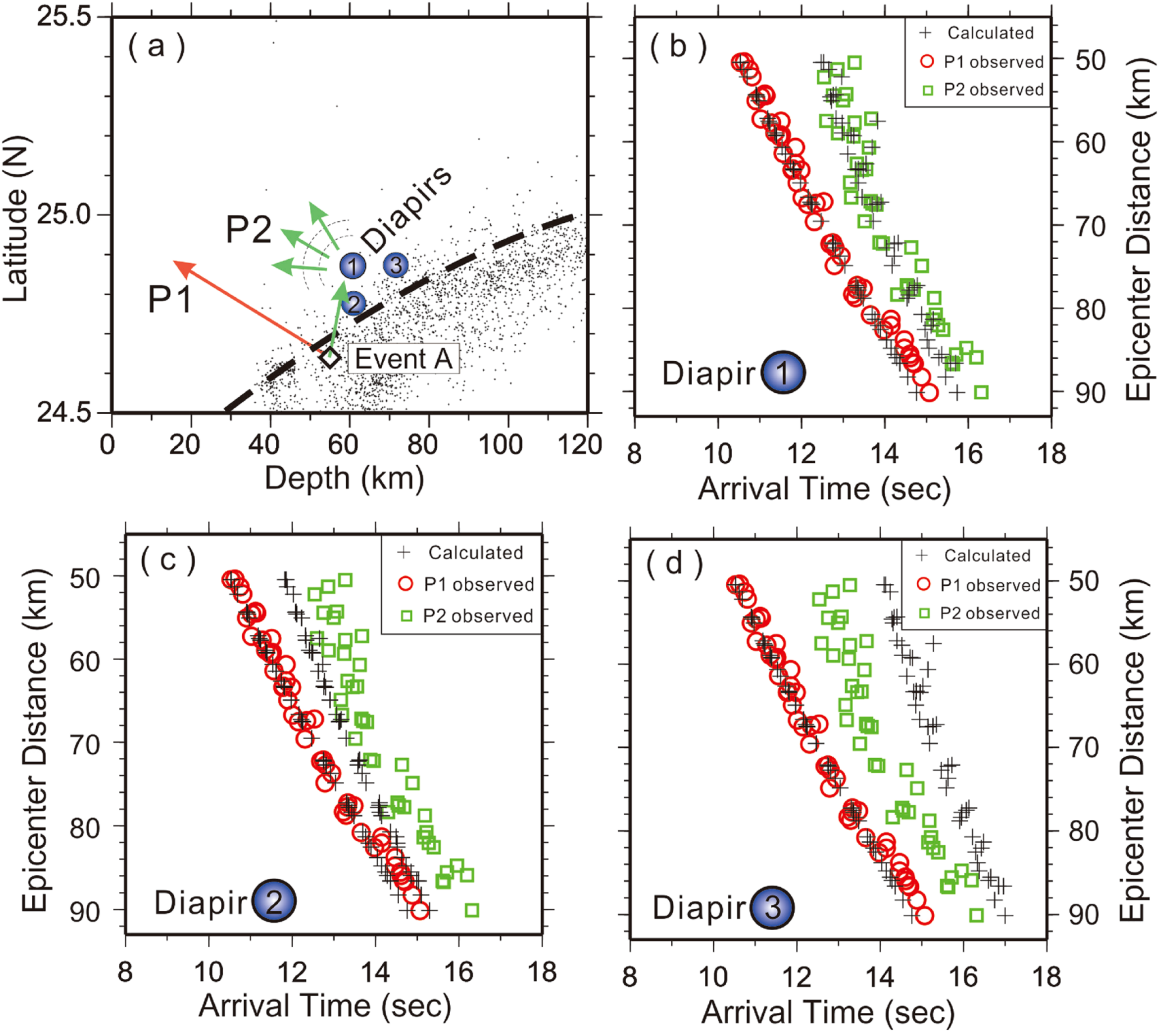
Sensitivity test for comparing diapirs at three locations (1, 2 and 3) separated by 10 km. (**a**) Locations of diapirs (blue circles), earthquake (diamond) and background seismicity (small dots). The comparison between the observed arrivals of P1 (red circles) and P2 (green squares), and their calculated arrivals (pluses) for the reflectors at locations 1, 2 and 3 are shown (**b**–**d**), respectively. This figure was created by Generic Mapping Tools (GMT version 4.5.2; URL: gmt.soest.hawaii.edu).

Although there are some location uncertainties, the results above are sufficient to distinguish the scatter obstacles from any known possible structures in and around the subduction zone because the general pattern of the P1 and P2 arrivals is very sensitive to the obstacle locations. To check the location sensitivity, for example, we calculate the travel-time differences between P1 and P2 arrivals for Event A as the scatter obstacle (Point 1) is shifted 10 km in the horizontal (Point 2) and vertical (Point 3) directions, respectively (Fig. 8). The results show significant travel-time deviations at Points 2 and 3, which are very close to the subducted slab. This means the location uncertainty of the scatter obstacle is less than a few kilometers and that the obstacle cannot be located at the subduction boundary. For the obstacles around depths of 90 km (Fig. 1), they are far away from the subduction slab and located within the mantle wedge where no significant structures have been reported. Similar sensitivity tests of locating scatter obstacles for Events B, C and D are shown in Figs. A1–A3. The results also show the locations of the obstacles are basically sensitive to the P2 arrivals, except Event D whose focal depth is largely deeper than that of the obstacles. Thus, the horizontal uncertainty of Event D might be slightly larger than that of other events.

## Discussion

This is the first seismic detection of scattering obstacles in the shallow mantle wedge just below active volcanoes in the Earth. Although some interesting structures have been imaged in the mantle wedge^11–15^, none have yet shown any seismic obstacles beneath active volcanoes. For instance, many seismic images obtained from tomographic inversion of either seismic velocity or attenuation often demonstrate some negative anomalies beneath the volcanic arc^11–13^. Those negative zones without any significant seismic boundary, likely representative of partial melting in the mantle wedge, are hardly scattering or reflecting seismic energy. The only case of detecting seismic reflections within the mantle wedge shows some nearly horizontal boundaries associated with a series of metasomatism within the mantle wedge^15^. Those boundaries are not only far away from the volcanic arc, but also too deep to be relative to typical magma generation at depths less than 100 km^39^. Therefore, the new discovery of seismic obstacles in the shallow mantle wedge beneath active volcanoes is entirely different from any previous seismic observations.

To explain the seismic observation of these scattering obstacles in the mantle wedge, a novel seismic model with mélange diapirs (or mantle-wedge plume) is preferentially considered here. Without any past seismic evidence, mélange diapirs have in fact been repeatedly proposed according to geochemical analyses and laboratory study^7–10^. In short, the concept is that mélanges first form along the interface between the subducted slab and the overlying mantle as the slab subducts. Blobs of low-density mélange, diapirs, then rise buoyantly from the surface of the subducting slab and are transported into the mantle wedge. Finally, the mélange diapirs partially melt to form magmas and gradually accumulate in magma reservoirs beneath the volcanic arc. The seismic detection of scattering obstacles here strongly support the model of mélange diapirs in the shallow mantle wedge based on the following considerations.

At first, strong seismic energy (P2) recorded at all seismic stations in the Formosa Array shows that the seismic boundary (or discontinuity) is significantly sharp. Such a sharp seismic boundary is wholly different from any vague seismic image of low velocity zones obtained from seismic tomography^11–13^, but might be representative of the chemical difference between the diapirs and surrounding mantle peridotite. Although the diapirs might be formed by a variety of compositions, the physical mixing by subducted sediments and crust, released fluids, and mantle peridotite is one of the most acceptable candidates that have been well discussed^7–10^.

Second, some small sphere-like obstacles are suitable for showing the diapirs (or mantle plumes) whose shape might be hypothesized as an upwelling of mélange from the top of the slab^4,5^. The spherical obstacles, representing upwelling diapirs, can very efficiently scatter seismic energy in all directions. Although it is currently unclear how large the scattering obstacles are, a radius of 1.0–1.5 km may be a reasonable estimation based on scaling analysis of instability growth rates^35^. The scattering obstacles might not be too large not only because they were not been detected by any seismic tomography in the past, but also because they might have problem to create all later P-waves (P2) recorded at the entire Formosa Array. Considering the diapir as a sphere with 1.0–1.5 km radius, thus, it will act as a scattering point because the radius of the diapir is significantly shorter than that (~ 8 km) of the incidence wavelength^38^. In other words, the diapir hit by the direct P-waves becomes as an activated source that radiates waves in all directions^36,37^. Therefore, a reasonable estimate of the obstacle radius may be less than a few kilometers. But such a small diapir is hardly detected by seismic tomography, and then other observations are needed before this can be later proven.

Third, the locations of the detected obstacles are basically consistent with the model of mélange diapirs just beneath two magma reservoirs at typical depths in northern Taiwan^9,10^. Based on map distributions (Fig. 1a), these scattering obstacles are separated into two groups. One is located beneath the TVG; the other is near Turtle Island. The location of scattering obstacles beneath the TVG almost overlies that of one magma reservoir detected by the S-wave shadow and P-wave delay^27^. Although the shallow group is very close to the subduction zone, it could not be at the interface between the subducted slab and the overlying mantle wedge according to the uncertainty estimation (Fig. 8). Similarly, the scattering obstacles near Turtle Island are just beneath another magma reservoir^28^. Although the obstacles are separated into two different depths, ~ 60 km and ~ 95 km, both are located just on top of the subducted slab. Overall, the scattering obstacles are located not only above the subducted slab but also just beneath the magma reservoirs. The depths of those scattering obstacles ranging between ~ 60 and ~ 95 km are basically similar to most typical diapir depths estimated from global subduction zones^35^. Besides, the spherical obstacles are formed by the mixed rocks of both the subducted crust and the overlying mantle around the depths greater than 50 km in the mantle wedge^5^. On the other hand, the flattened diapirs are formed by unmixed mantle at the depths between 30 and 50 km. Therefore, sphere-like scattering obstacles may be suitable for representing mélange diapirs beneath two active volcanoes in northern Taiwan.

Finally, previous isotope analyses of volcanic rocks at the TVG show that the mixing of subducted sediments with the lithosphere mantle is required. Based on a mixing calculation of the Nd–Sr isotope composition^39^, 2% subducted sediments added to the mantle are enough to explain the observations of ^87^Sr/^86^Sr (0.7038–0.7048) and ^143^Nd/^144^Nd (0.5126–0.5128) at volcanic rocks in the TVG. This chemical feature is extremely similar to those observed at other typical volcanic arcs, such as Marianas, Tonga, and Lesser Antilles^6^, where the mantle and sediments are mixing in the volcanic rocks. Furthermore, the Nb/La ratio of ~ 0.5 obtained from volcanic rocks in the TVG indicates that they are derived from the lithosphere mantle altered by subduction-related metasomatism^40^. Such a result can be sufficiently distinguished from an asthenospheric source, MORB (mid-ocean ridge basalt), that has an Nb/La ratio greater than 1.0^41,42^. The isotope data consistently demonstrate that volcanic rocks in the TVG result from the mixing of subducted sediments or slab with the lithosphere mantle.

In summary, this study is the first to report seismic scattering obstacles in the shallow mantle wedge of the subduction zone (Fig. 1c). For the first order approximation, those scattering obstacles might be simply considered as a sphere with a radius of ~ 1 km even though the exact shape and size are still not well constrained. These obstacles might be delegated to the mélange diapirs that are physically mixed by the subducted sediments and crust, released fluids, and mantle peridotite. Although the mélange diapirs are preferentially considered here, other possibility, such as formed by ascending melts or slab material only, might not be totally excluded. Some further studies from the seismic data recorded by the Formosa Array might be helpful to distinguish the content of the scattering obstacles in the near future. Finally, these obstacles might be directly associated with magma generation in the uppermost mantle because they are identified just beneath two active volcanoes of the TVG and Turtle Island in northern Taiwan.

## Methods

We have carefully assessed the P1 and P2 arrivals from the seismograms recorded at the Formosa Array in northern Taiwan. Because of near-vertical incidences, the P1 arrivals are clearly detected on the seismograms generated by local mantle-depth earthquakes. Although P2 arrivals might be contaminated by some scattering energy from P1 due to possible heterogeneity in the crust, the consistent energy of P2 arrivals can be identified when we check the seismograms at one station with those of adjacent stations within ~ 5 km. In Fig. 3, for example, the arrivals of P2 are consistently observed along the dashed lines at Stations CT01–CT05, CT06–CT09, and KE15–KE18, at which the background noises are strong in the Taipei metropolitan area. For the typical Station CT02, where background noises are strong, the P1 and P2 arrivals can be detected not only at individual stations but also aligned with adjacent stations (CT01 and CT03). In other words, a high-density seismic array significantly improves the detection of seismic energy coming from the same source.

A conventional trial and error method is employed to find the most suitable scattering obstacle for each earthquake. Since the general trend of P2 arrivals often follow a hyperbolic curve, it follows that the P2 arrivals are originating from the hypocenter, but scattered by other structures. To fit the observations, we simply calculate the travel-time of P1 and P2 in a layer velocity model of the upper mantle and overlying crust. The mantle velocity is given by 8.0 km/s and the crustal velocity is assumed to be an averaged value of 6.5 km/s in Taiwan^43^. The travel-time of P1 is directly calculated from an earthquake to all seismic stations. The travel-time of P2 is the summation of two parts: one is the travel-time from the earthquake to the scattering obstacle and the other is from the scattering obstacle to the seismic stations. For successfully scattering seismic energy at any incident direction, we simply assume that the 1st order approximation of the scattering obstacle is a sphere with a radius of 1 km based on the scaling analysis of instability growth rates^35^.

In fact, the location of the scattering obstacle can be roughly estimated from P2 arrivals recorded at the Formosa Array. Since the Formosa Array has high-density seismic stations with a spacing of ~ 5 km, the possible scattering obstacle will be close to the station at which the P2 arrival is faster than any other seismic station. The depth of the scattering obstacle can also be estimated from the delay time (***dt***) between the seismic stations at different distances from the scattering source if the scattering is inside the Formosa Array (i.e., 34). The following equation shows the delay time (***dt***) at seismic stations with horizontal distances between ***x*** and* 0* from the source:

1 $${\varvec{d}}{\varvec{t}}=\frac{\sqrt{{{\varvec{x}}}^{2}+{{\varvec{z}}}^{2}}}{{\varvec{v}}}-\boldsymbol{ }\frac{{\varvec{z}}}{{\varvec{v}}\boldsymbol{ }},$$

in which ***x*** is the horizontal distance from the source, ***z*** is the source depth, and* v* is the P-wave velocity. Thus, reading delay time (***dt***) at a horizontal distance (***x***), we can calculate the depth (***z***) value by given ***v*** = 7.8 km/s. This estimated location from the P2 arrivals provides an initial position for further calculations.

Through some trial and error processes, the final location of the suitable scattering obstacle is effectively obtained when most of the calculated and observed times are comparable (Figs. 4, 5, 6, 7). Although there are some small outliers between the calculations and observations, the result might be acceptable without considering both the possible reading errors produced by unexpected noises at each seismic station and calculated uncertainties due to the 1D model and earthquake location. Even so, estimated uncertainty of the scattering locations is limited to within a few kilometers due to the small number of outliers (less than 1.0 s) at some stations.

## Supplementary Information

Supplementary Figures.
