# Supplementary material for: Mantle wedge diapirs detected by a dense seismic array in Northern Taiwan
Source: Sci Rep. 2021 Jan 15;11:1561. doi: 10.1038/s41598-021-81357-7 (PMC7810894; doi:10.1038/s41598-021-81357-7)
Supplement: Supplementary file 1 — Supplementary Figures. [file 41598_2021_81357_MOESM1_ESM.docx]

**Mantle Wedge Diapirs Detected by a Dense Seismic Array in Northern Taiwan**

**(Short title: Seismic Detection of Mantle Wedge Diapirs)**

Cheng-Horng Lin^1, 2, 3, 4,*^, Min-Hung Shih^1, 3^ and Ya-Chuan Lai^2, 3^

1. Institute of Earth Sciences, Academia Sinica, Taipei, Taiwan
2. National Center for Research on Earthquake Engineering, National Applied Research Laboratories, Taipei, Taiwan
3. Taiwan Volcano Observatory at Tatun, Taipei, Taiwan
4. Dept. of Geosciences, National Taiwan University, Taipei, Taiwan

(*) Corresponding author:

Cheng-Horng Lin

P O Box 1-55, Nankang, Taipei, Taiwan

Email: lin@earth.sinica.edu.tw

Tel: 886-2-27839910 ext. 1521

Fax: 886-2-27839159

Submitted to ***Scientific Reports*** on August 23, 2020

1^st^ revised on Nov. 18, 2020

2^nd^ revised on December 29, 2020

**Supplementary information:**

Three supplementary figures (Figs. A1-A3) are added here for showing the sensitivity test for comparing the scattering obstacle at three locations (1, 2 and 3) for Events B, C and D. Similar to the results in Fig. 8, the locations of the scattering obstacles are basically sensitive to the P2 arrivals except Event D whose focal depth is largely deeper than that of the obstacles.


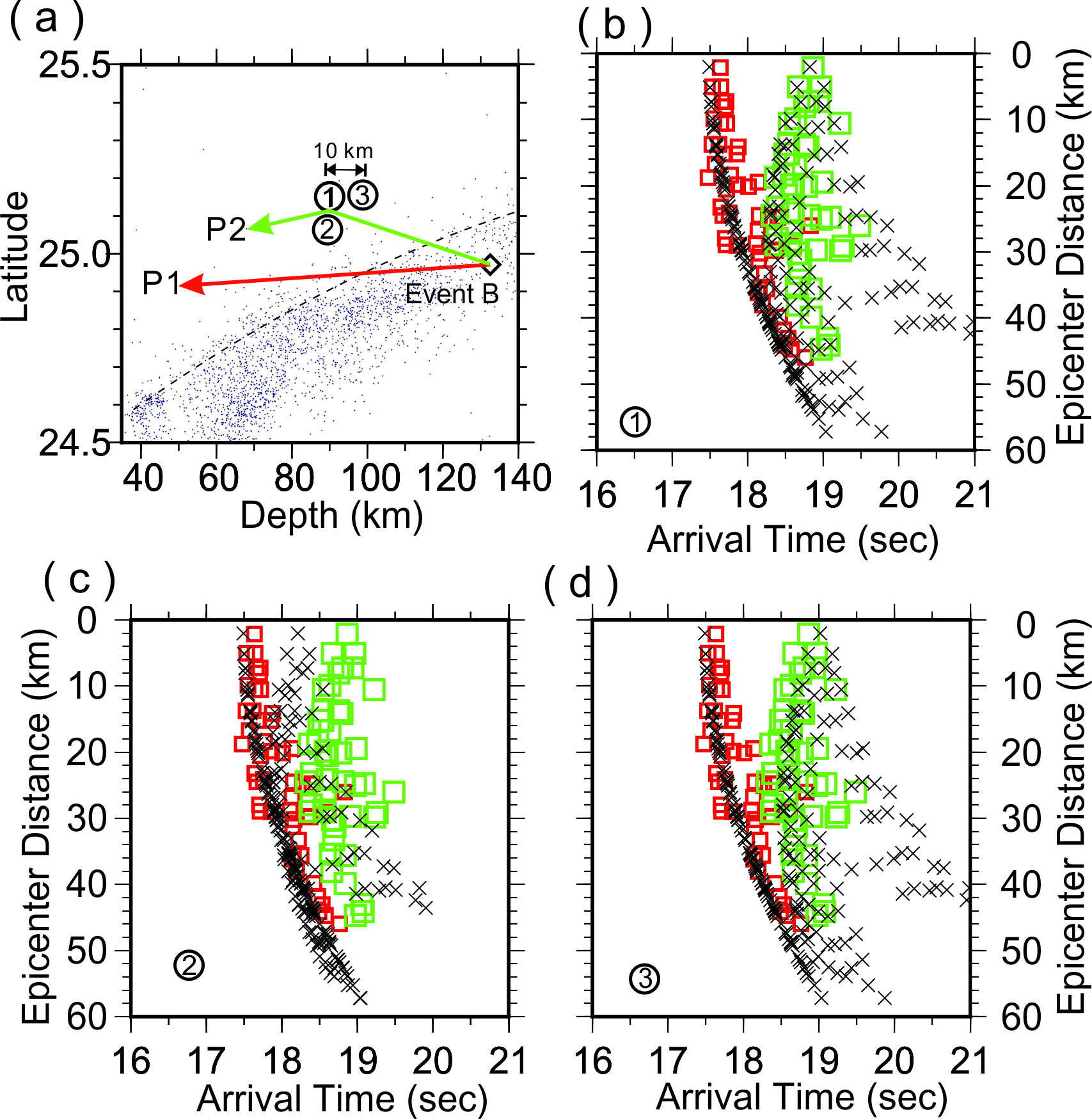


Fig. A1 Sensitivity test of Event B for comparing diapirs at three locations (1, 2 and 3) separated by 10 km. (a) Locations of three diapirs (circles), earthquake (diamond) and background seismicity (small dots). The comparison between the observed arrivals of P1 (red circles) and P2 (green squares), and their calculated arrivals (crosses) for the diapirs at locations 1, 2 and 3 are shown (b), (c) and (d), respectively. This figure was created by Generic Mapping Tools (GMT version 4.5.2; URL: gmt.soest.hawaii.edu).


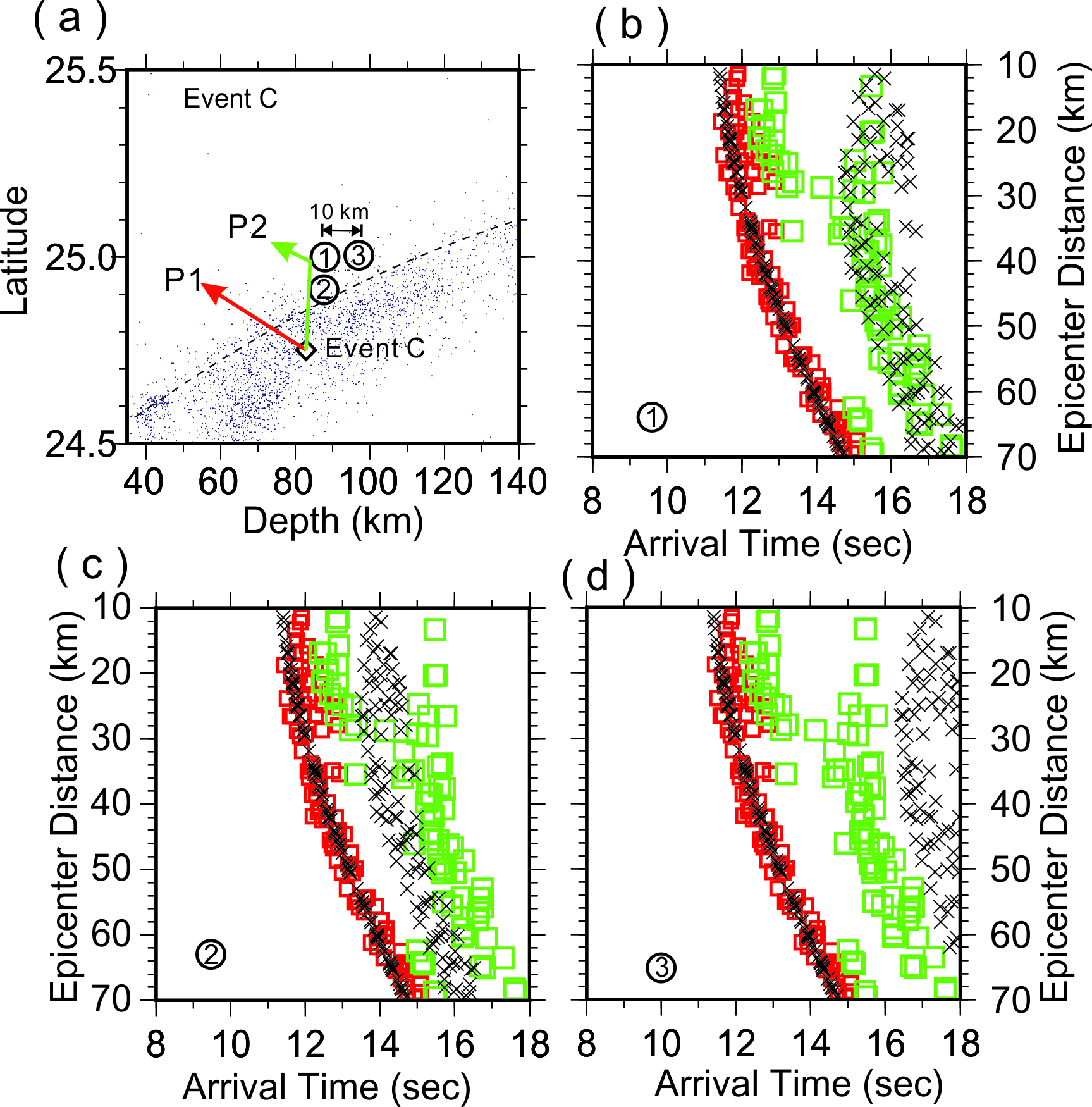


Fig. A2 Sensitivity test of Even C for comparing diapirs at three locations (1, 2 and 3) separated by 10 km. (a) Locations of three diapirs (circles), earthquake (diamond) and background seismicity (small dots). The comparison between the observed arrivals of P1 (red circles) and P2 (green squares), and their calculated arrivals (crosses) for the diapirs at locations 1, 2 and 3 are shown (b), (c) and (d), respectively. This figure was created by Generic Mapping Tools (GMT version 4.5.2; URL: gmt.soest.hawaii.edu).


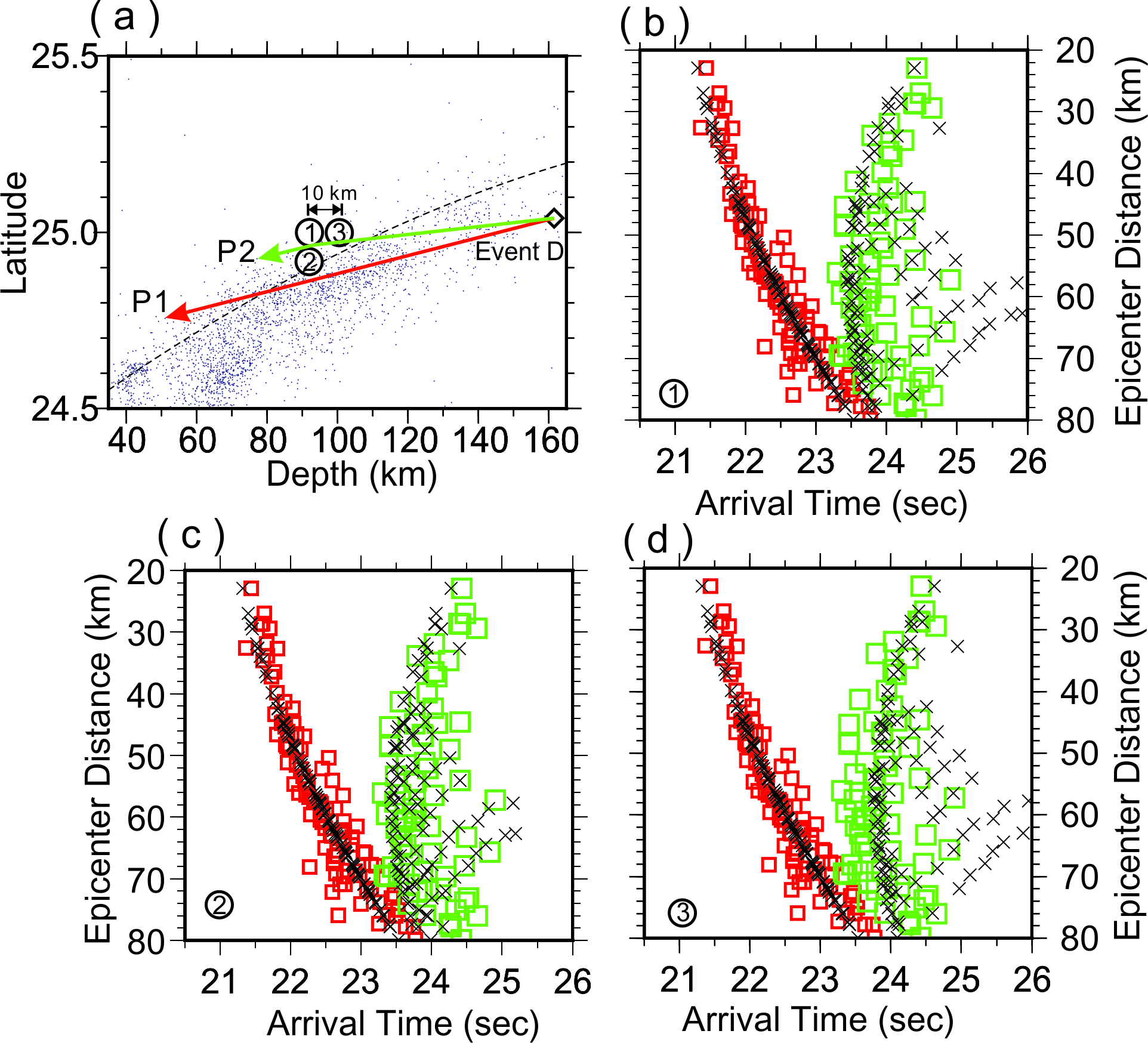


Fig. A3 Sensitivity test of Event D for comparing diapirs at three locations (1, 2 and 3) separated by 10 km. (a) Locations of three diapirs (circles), earthquake (diamond) and background seismicity (small dots). The comparison between the observed arrivals of P1 (red circles) and P2 (green squares), and their calculated arrivals (crosses) for the diapirs at locations 1, 2 and 3 are shown (b), (c) and (d), respectively. This figure was created by Generic Mapping Tools (GMT version 4.5.2; URL: gmt.soest.hawaii.edu).
